# Supplementary material for: Gene-level metagenomic architectures across diseases yield high-resolution microbiome diagnostic indicators
Source: Nat Commun. 2021 May 18;12:2907. doi: 10.1038/s41467-021-23029-8 (PMC8131609; doi:10.1038/s41467-021-23029-8)
Supplement: Supplementary file 1 — Supplementary Information [file 41467_2021_23029_MOESM1_ESM.pdf]

Supplementary Information

Gene-level metagenomic architectures across diseases yield high-resolution microbiome diagnostic indicators

Braden T Tierney[1,2,3,4], Yingxuan Tan[1], Aleksandar D Kostic[2,3,4+], Chirag J Patel[1+]
[+]co-corresponding author

Corresponding authors:
Chirag J Patel
chirag_patel@hms.harvard.edu

Aleksandar D Kostic
Aleksandar.Kostic@joslin.harvard.edu

[1]Department of Biomedical Informatics, Harvard Medical School, Boston, MA 02115, USA
[2]Section on Pathophysiology and Molecular Pharmacology, Joslin Diabetes Center, Boston, MA 02215, USA
[3]Section on Islet Cell and Regenerative Biology, Joslin Diabetes Center, Boston, MA 02215, USA
[4]Department of Microbiology, Harvard Medical School, Boston, MA 02115, USA

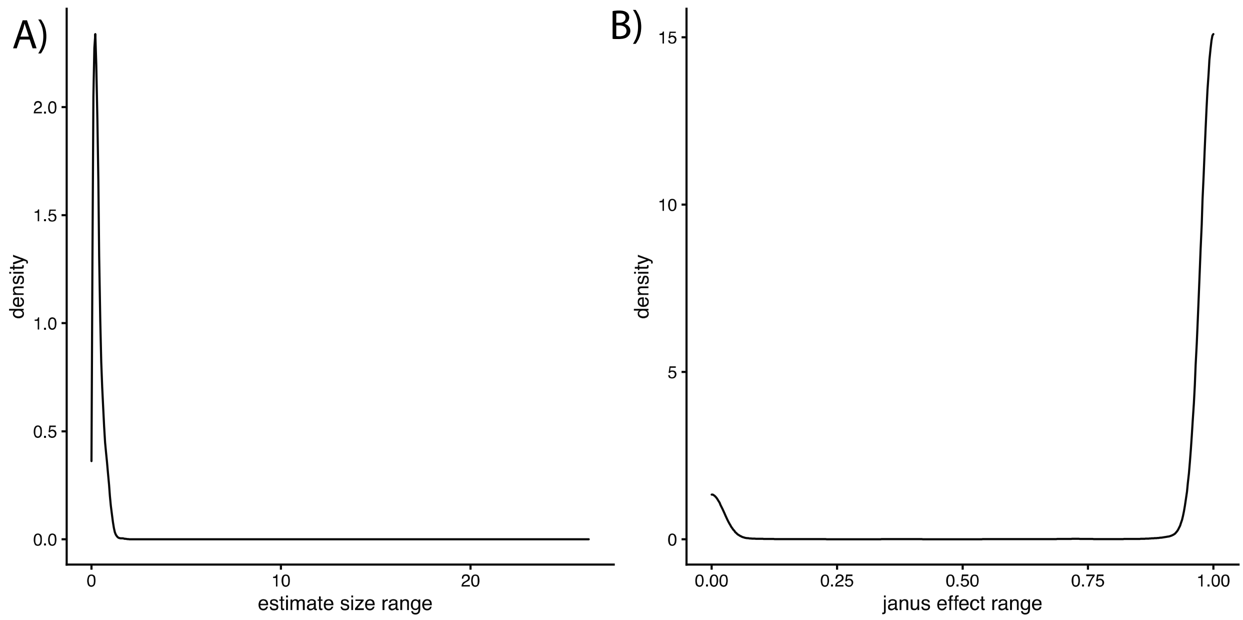

Supplementary Figure 1: **Modeling vibration of effects, overall results.** The distribution of A) estimate ranges, and B) Janus effects across all regressions for the disease-variable in our regressions.

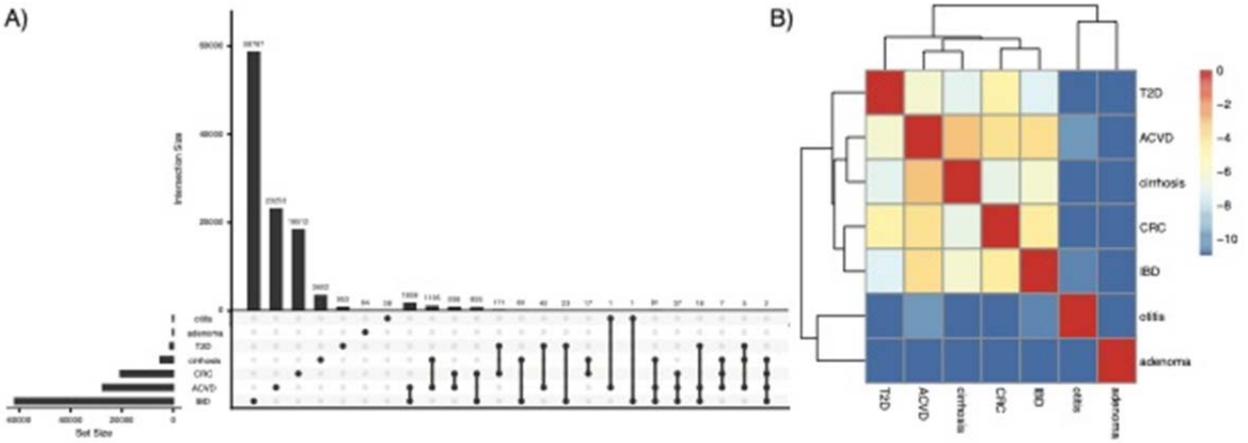

Supplementary Figure 2: **Overall architectures.** The overall similarity and differences between microbiome disease structures across all data modalities. A) The combined species, pathways, and gene families associated with each phenotype and the overlap therein. B) The natural log of Jaccard similarity between pairwise combinations of phenotypes (See Methods).

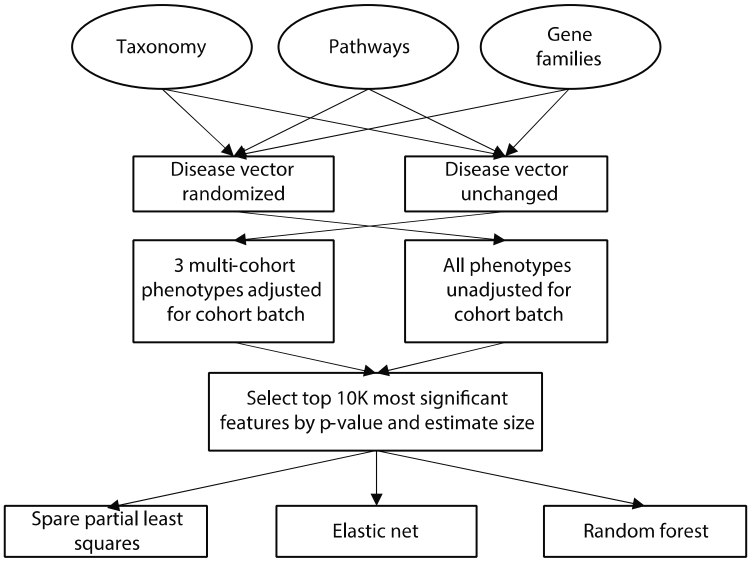

Supplementary Figure 3: **Benchmarking strategy.** We compared our modeling strategy (consisting of association, meta-analysis, and vibration of effects) to a number of other potential methods that are frequently used in microbiome studies. Specifically, we aimed to identify if 1) the variable importance/estimate sizes were similar between different methods and 2) if a randomization test (randomizing the disease vector) showed that our or similar approaches would yield large numbers of false positive results. As such, for each data type (top row), and with the disease vector randomized/non-randomized, we computed association for each feature using a univariate linear regression, or, where possible, linear regression adjusted for cohort. To avoid failure due to computational complexity, we then selected the top 10 thousand most significant features and ran the methods in the bottom row, comparing the output to all other steps.

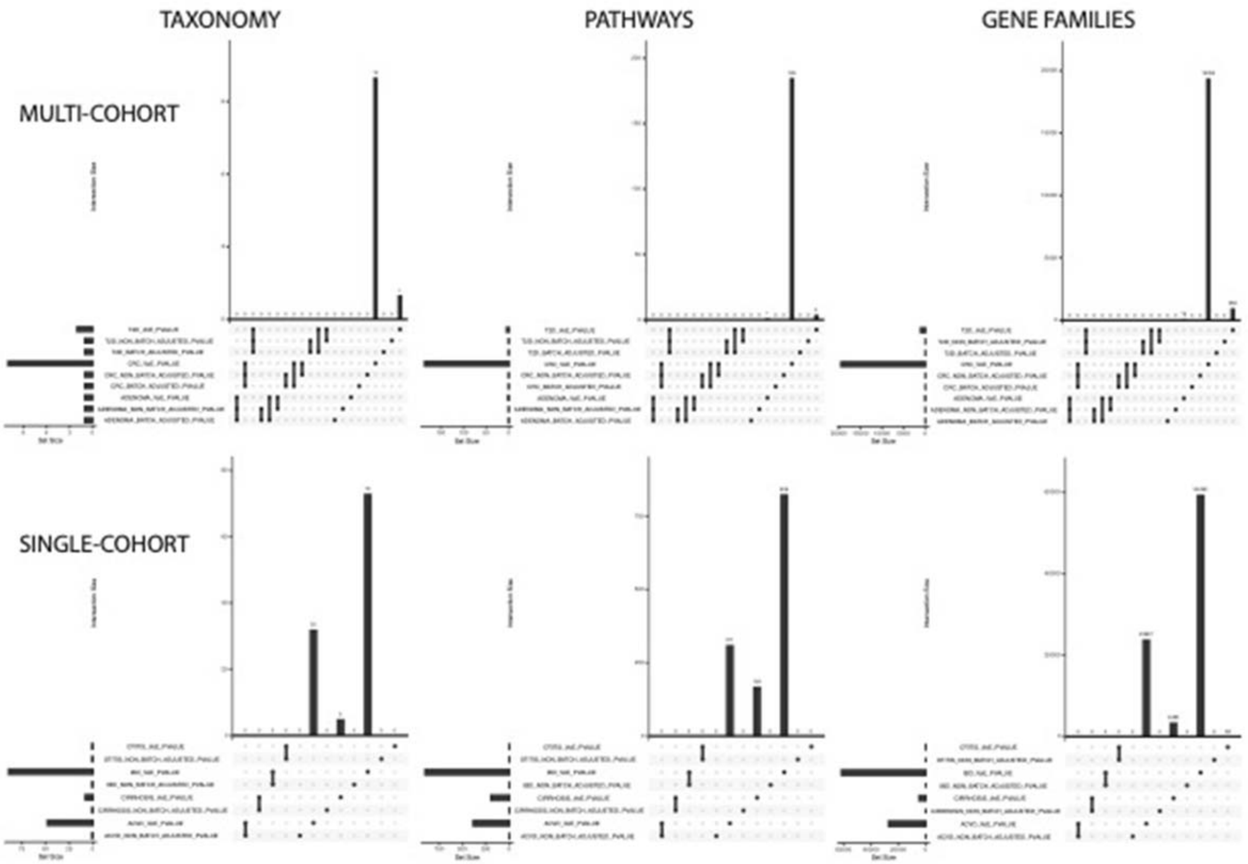

Supplementary Figure 4: **Permutation experiment.** As a negative control, for the modeling strategies described in Supplementary Figure 3, we randomized the disease variable and identified p-values where possible (from the linear models) and compared overlaps in statistical significance between our initial output and other linear modeling methods. We took robust (by modeling VoE) and False-Discovery-Rate (FDR < 0.05) significant features for the initial maximal model associations in our pipeline and compared those to features that had a nominal p-value less than the p-value equivalent to FDR = 0.05 in our initial approach.

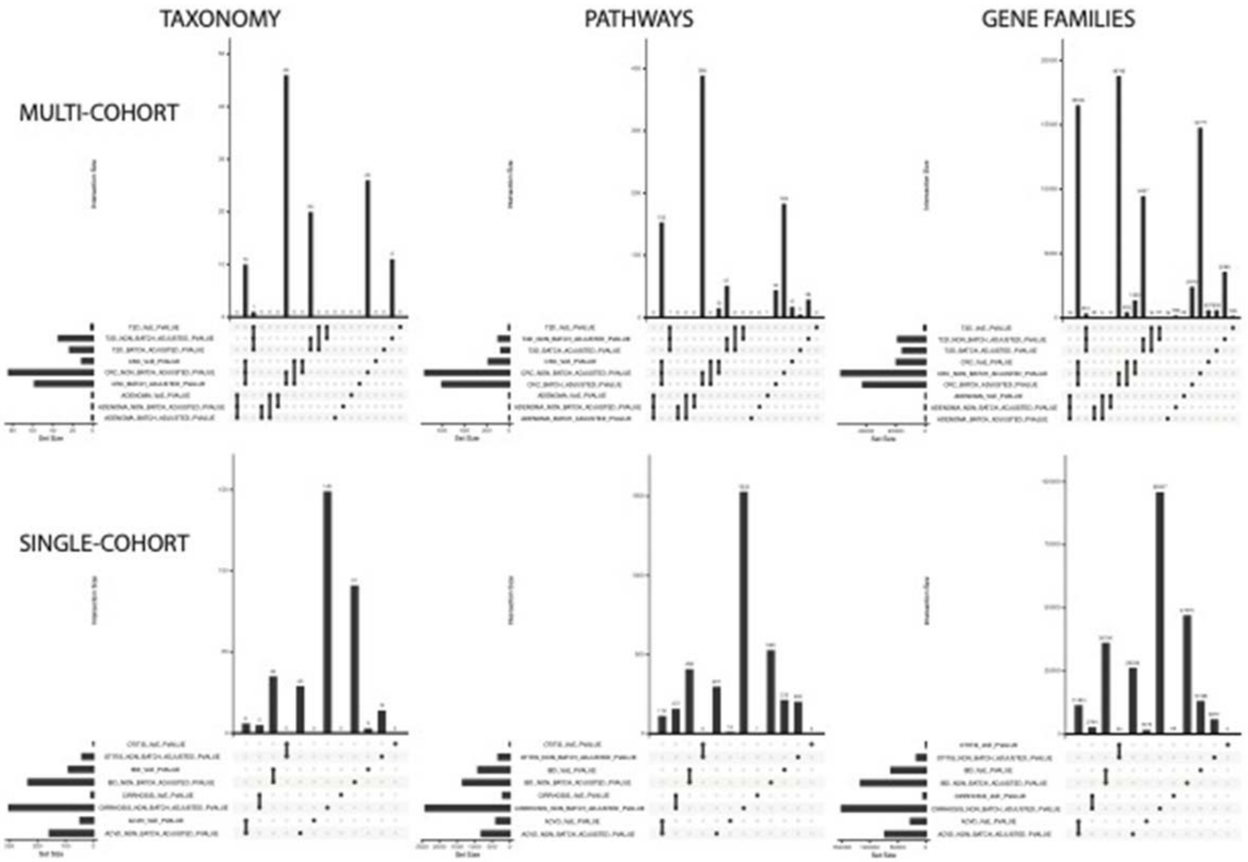

Supplementary Figure 5: **Comparing modeling strategies.** For the modeling strategies described in Supplementary Figure 3, we identified p-values where possible (from the linear models) and compared overlaps in statistical significance between our initial output and other linear modeling methods. We took robust (by modeling VoE) and False-Discovery-Rate (FDR < 0.05) significant features for the initial maximal model associations in our pipeline and compared those to features that had a nominal p-value less than the p-value equivalent to FDR = 0.05 in our initial approach.

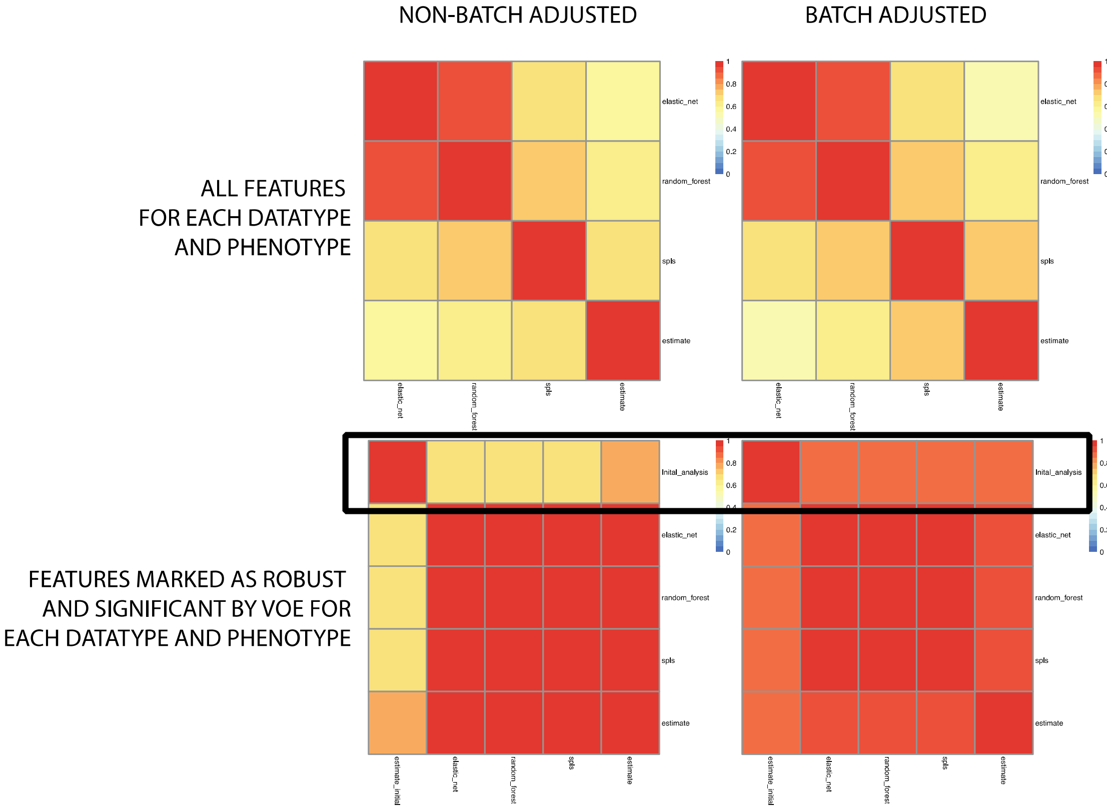

Supplementary Figure 6: **Concordance between regression methods.** We measured the estimated the concordance in feature ranking (e.g. relative importance or absolute value of beta-coefficient) for each method we compared for (top row) all features used in the comparison analysis for each datatype and phenotype and (bottom row) only the features deemed statistically significant and robust by our initial pipeline. Columns correspond to if the linear model used to select the 10 thousand features for variable selection analysis was adjusted for cohort batch or not. The black box indicates the correlation between the ranking of the absolute value of beta-coefficients from our pipeline for robust features (derived from the maximal models) and the other methods.
