# Supplementary material for: Gene-level metagenomic architectures across diseases yield high-resolution microbiome diagnostic indicators
Source: Nat Commun. 2021 May 18;12:2907. doi: 10.1038/s41467-021-23029-8 (PMC8131609; doi:10.1038/s41467-021-23029-8)
Supplement: Supplementary file 3 — Description of Supplementary Data Files [file 41467_2021_23029_MOESM3_ESM.docx]

**Description of Supplementary Data Files**

Gene-level metagenomic architectures across diseases yield high-resolution microbiome diagnostic indicators

Braden T Tierney^1,2,3,4^, Yingxuan Tan^1^, Aleksandar D Kostic^2,3,4+^, Chirag J Patel^1+^

^+^co-corresponding author

Corresponding authors:

Chirag J Patel

[chirag_patel@hms.harvard.edu](mailto:chirag_patel@hms.harvard.edu)

Aleksandar D Kostic

[Aleksandar.Kostic@joslin.harvard.edu](mailto:Aleksandar.Kostic@joslin.harvard.edu)

^1^Department of Biomedical Informatics, Harvard Medical School, Boston, MA 02115, USA

^2^Section on Pathophysiology and Molecular Pharmacology, Joslin Diabetes Center, Boston, MA 02215, USA

^3^Section on Islet Cell and Regenerative Biology, Joslin Diabetes Center, Boston, MA 02215, USA

^4^Department of Microbiology, Harvard Medical School, Boston, MA 02115, USA

File name: Supplementary Data 1

Description: Cohort summary statistics and models used in initial regression strategy.

File name: Supplementary Data 2

Description: Glossary of terms.

File name: Supplementary Data 3

Description: Number of significant features per disease at each step in pipeline and list of FDR-significant and robust species, pathways, and gene families.

File name: Supplementary Data 4

Description: List of genes used in comparative architecture analysis for Figure 5.
