# Supplementary material for: Gene-level metagenomic architectures across diseases yield high-resolution microbiome diagnostic indicators
Source: Nat Commun. 2021 May 18;12:2907. doi: 10.1038/s41467-021-23029-8 (PMC8131609; doi:10.1038/s41467-021-23029-8)
Supplement: Supplementary file 8 — Reporting Summary [file 41467_2021_23029_MOESM8_ESM.pdf]

nature research

Corresponding author(s): Chirag J Patel, Aleksandar D Kostic

Last updated by author(s): Mar 31, 2021

Reporting Summary

Nature Research wishes to improve the reproducibility of the work that we publish. This form provides structure for consistency and transparency in reporting. For further information on Nature Research policies, see our Editorial Policies and the Editorial Policy Checklist.

Statistics

For all statistical analyses, confirm that the following items are present in the figure legend, table legend, main text, or Methods section.

n/a	Confirmed	
[ ]	[x]	The exact sample size (*n*) for each experimental group/condition, given as a discrete number and unit of measurement
[ ]	[x]	A statement on whether measurements were taken from distinct samples or whether the same sample was measured repeatedly
[x]	[ ]	The statistical test(s) used AND whether they are one- or two-sided<br>*Only common tests should be described solely by name; describe more complex techniques in the Methods section.*
[ ]	[x]	A description of all covariates tested
[ ]	[x]	A description of any assumptions or corrections, such as tests of normality and adjustment for multiple comparisons
[x]	[ ]	A full description of the statistical parameters including central tendency (e.g. means) or other basic estimates (e.g. regression coefficient) AND variation (e.g. standard deviation) or associated estimates of uncertainty (e.g. confidence intervals)
[ ]	[x]	For null hypothesis testing, the test statistic (e.g. *F*, *t*, *r*) with confidence intervals, effect sizes, degrees of freedom and *P* value noted<br>*Give P values as exact values whenever suitable.*
[x]	[ ]	For Bayesian analysis, information on the choice of priors and Markov chain Monte Carlo settings
[ ]	[x]	For hierarchical and complex designs, identification of the appropriate level for tests and full reporting of outcomes
[x]	[ ]	Estimates of effect sizes (e.g. Cohen's *d*, Pearson's *r*), indicating how they were calculated

Our web collection on statistics for biologists contains articles on many of the points above.

Software and code

Policy information about availability of computer code

Data collection	Raw microbiome data was accessed from the curatedMetagenomicData (https://waldronlab.io/curatedMetagenomicData/) package and can be downloaded with the package therein. Additional datasets used for validation can be downloaded and accessed at ENA accession PRJEB27928 and https://ibdmdb.org/tunnel/public/summary.html, with additional metadata for the former being available at https://github.com/waldronlab/curatedMetagenomicDataCuration/blob/master/inst/curated/WirbelJ_2018/WirbelJ_2018_metadata.tsv.
Data analysis	The majority of the analysis, unless otherwise specified, was carried out in R >V3.6.0. For processing, we used the Tidyverse V1.3.0. The package curatedMetagenomicData V1.14.1 was used for data aggregation. All microbiome data provided by said package was analyzed (be its developers) with HUMAnn2 V0.7.1. We computed shannon diversity with the Vegan package V2.5.6 and computed meta-analyses with metafor V2.4.0. Taxonomizr V0.5.0 was used to process NCBI taxon data. ETE3 V3.1.2 was used (with Python V3.7.4) to build phylogenetic trees. We fit additional models using the caret V6.086 package for random forests and the elastic net and the mixOmics V6.12.2 package for sPLS. We generated all plots with R’s ggplot2 package V3.3.2, the exception being forest plots, which we made with the meta package V.13.0.

For manuscripts utilizing custom algorithms or software that are central to the research but not yet described in published literature, software must be made available to editors and reviewers. We strongly encourage code deposition in a community repository (e.g. GitHub). See the Nature Research guidelines for submitting code & software for further information.

Data

Policy information about availability of data

All manuscripts must include a data availability statement. This statement should provide the following information, where applicable:

- Accession codes, unique identifiers, or web links for publicly available datasets
- A list of figures that have associated raw data
- A description of any restrictions on data availability

All relevant datasets are publicly available. Those used in the initial analysis can be downloaded from the R package associated with curatedMetagenomicData.34 We used this package's compilation of the data into single dataframes. The dataset information can be found in the "combined_metadata" file available with the package release. Additional datasets used for validation can be downloaded and accessed at ENA accession PRJEB27928 and https://ibdmdb.org/tunnel/public/summary.html, with additional metadata for the former being available at https://github.com/waldronlab/curatedMetagenomicDataCuration/blob/master/inst/curated/WirbelJ_2018/WirbelJ_2018_metadata.tsv. The UniRef90/UniProtKB database, used for identifying gene-level NCBI taxonomic identifiers, can be found at https://www.uniprot.org/.

Field-specific reporting

Please select the one below that is the best fit for your research. If you are not sure, read the appropriate sections before making your selection.

☒ Life sciences ☐ Behavioural & social sciences ☐ Ecological, evolutionary & environmental sciences

For a reference copy of the document with all sections, see nature.com/documents/nr-reporting-summary-flat.pdf

Life sciences study design

All studies must disclose on these points even when the disclosure is negative.

Sample size	Sample sizes of each individual cohort is documented in the manuscript. We chose to use all of the samples present in these cohort datasets, with the assumption that maximizing sample size would increase our statistical power as much as possible.
Data exclusions	No data were excluded.
Replication	We reproduced our findings for two of our diseases of interest in external, also publicly available cohorts.
Randomization	This is not relevant to our study. They were all observational and did not perform any randomization.
Blinding	Blinding was not relevant to our study as this was a discovery-based analysis. We were not attempting to test the clinical impact of a particular intervention, and following the standard in the microbiome field did not find it necessary to blind our analysts.

Reporting for specific materials, systems and methods

We require information from authors about some types of materials, experimental systems and methods used in many studies. Here, indicate whether each material, system or method listed is relevant to your study. If you are not sure if a list item applies to your research, read the appropriate section before selecting a response.

Materials & experimental systems

n/a	Involved in the study
☒	☐ Antibodies
☒	☐ Eukaryotic cell lines
☒	☐ Palaeontology and archaeology
☒	☐ Animals and other organisms
☒	☐ Human research participants
☒	☐ Clinical data
☒	☐ Dual use research of concern

Methods

n/a	Involved in the study
☒	☐ ChIP-seq
☒	☐ Flow cytometry
☒	☐ MRI-based neuroimaging
